# The Cold-Inducible RNA-Binding Protein (CIRP) Level in Peripheral Blood Predicts Sepsis Outcome

**DOI:** 10.1371/journal.pone.0137721

**Published:** 2015-09-11

**Authors:** Yanyan Zhou, Haiyun Dong, Yanjun Zhong, Jia Huang, Jianlei Lv, Jinxiu Li

**Affiliations:** Intensive Care Unit, The Second Xiangya Hospital, Central South University, Changsha, Hunan, China; University of Leicester, UNITED KINGDOM

## Abstract

**Objectives:**

Sepsis is a lethal and complex clinical syndrome caused by infection or suspected infection. Cold-inducible RNA-binding protein (CIRP) is a widely distributed cold-shock protein that plays a proinflammatory role in sepsis and that may induce organ damage. However, clinical studies regarding the use of CIRP for the prognostic evaluation of sepsis are lacking. The purpose of this research was to investigate the prognostic significance of peripheral blood concentrations of CIRP in sepsis. Sepsis was assessed using several common measures, including the Acute Physiology and Chronic Health Evaluation II (APACHE II) score; the Sepsis-related Organ Failure Assessment (SOFA) score; the lactate, serum creatinine, and procalcitonin (PCT) levels; the white blood cell (WBC) count; and the neutrophil ratio (N%).

**Design:**

Sixty-nine adult patients with sepsis were enrolled in this study. According to the mortality data from the hospital, 38 patients were survivors, and 31 were nonsurvivors. The plasma levels of the biomarkers were measured and the APACHE II and SOFA scores were calculated within 24 hours of patient enrollment into our study. The CIRP level was measured via ELISA.

**Results:**

The plasma level of CIRP was significantly higher in the nonsurvivors than in the survivors (median (IQR) 4.99 (2.37–30.17) ng/mL and 1.68 (1.41–13.90) ng/mL, respectively; *p* = 0.013). The correlations of the CIRP level with the APACHE II score (r = 0.248, *p* = 0.040, n = 69), the SOFA score (r = 0.323, *p* = 0.007, n = 69), the serum creatinine level (r = 0.316, *p* = 0.008, n = 69), and the PCT level (r = 0.282, *p* = 0.019, n = 69) were significant. Receiver operator characteristic (ROC) curve analysis showed that the area under the ROC curve (AUC) for the CIRP level was 0.674 (*p* = 0.013). According to Cox proportional hazards models, the CIRP level independently predicts sepsis mortality. When the CIRP level in the peripheral blood increased by 10 ng/mL, the mortality risk increased by 1.05-fold (*p* = 0.012). Thus, the CIRP level reflects the degree of renal injury but does not predict the severity of sepsis or organ damage.

**Conclusion:**

An elevated plasma concentration of CIRP was significantly associated with poor prognosis among patients with sepsis. Therefore, CIRP is a potential predictor of sepsis prognosis.

## Introduction

Sepsis is a type of systemic inflammatory response syndrome (SIRS) that is secondary to documented or suspected infection [1]. According to several reports published in the *New England Journal of Medicine*, the 90-day mortality of severe sepsis/septic shock varies from 18.7% to 44% [2–6]. Data from the Healthcare Cost and Utilization Project in the United States in 2011 revealed that sepsis was the most costly disease in hospitalized patients. In that study, at discharge, 59% of patients with sepsis experienced major complications or comorbidities, whereas only 27.5% of sepsis patients experienced no complications or comorbidities [7]. Biomarkers, including C-reactive protein, procalcitonin (PCT) and interleukin-6, are commonly used in the clinical setting to evaluate the severity of sepsis [8–10].

In 1997, cold-inducible RNA-binding protein (CIRP), the first cold-shock protein and a member of the glycine-rich RNA-binding protein family, was found in mammalian cells [11]. CIRP, a multifunction protein, plays an important role in many biological activities, such as tumorigenesis, hypothermic injury, UV-C irradiation-related inflammation, clock gene regulation, and limb regeneration [12–19]. In patients experiencing hemorrhagic shock, the plasma CIRP concentrations are increased. Animal studies have confirmed that CIRP is up-regulated and is released into the circulation during hemorrhage and sepsis. Furthermore, many studies have demonstrated that CIRP is a pro-inflammatory cytokine that can induce various inflammatory reactions [20–23]. However, clinical studies are lacking regarding the prognostic utility of CIRP for the evaluation of sepsis. The purpose of this research was to investigate the prognostic significance of the peripheral blood level of CIRP in patients with sepsis. Sepsis was assessed using common measures, including the Acute Physiology and Chronic Health Evaluation II (APACHE II) score; the Sepsis-related Organ Failure Assessment (SOFA) score; the lactate, serum creatinine, and PCT levels; the white blood cell (WBC) count; and the neutrophil ratio (N%).

## Material and Methods

### Participants

This was a clinical observational study conducted at the Second Xiangya Hospital of Central South University from November 2013 to June 2014. Sixty-nine critically ill adult patients with sepsis in the general intensive care unit (ICU) were enrolled in our study [1, 24]. According to the mortality records from the hospital, there were 38 survivors and 31 nonsurvivors. The predisposing conditions included severe pulmonary infection, severe acute pancreatitis, digestive tract perforation, postoperative infection following organ transplantation, intracranial infection, aspiration, bladder perforation, trauma, intestinal obstruction, gas gangrene, and perinephric abscesses. All of the enrolled patients were followed until death in the hospital or discharge and were then defined as nonsurvivors or survivors. The definition of severe sepsis is sepsis combined with sepsis-induced organ dysfunction or tissue hypoperfusion [1, 24]. Severe sepsis and septic shock are evidence of sepsis-induced deterioration and suggest a poorer prognosis. The APACHE II score was used as an additional assessment of the severity of sepsis, and the SOFA score was used for the assessment of organ failure [25, 26]. To analyze the difference between severe sepsis or septic shock patients and septic patients without severe sepsis or septic shock, we compared the correlations between the CIRP level and both the APACHE II score and the SOFA score. The exclusion criteria included age less than 18 years, pregnancy and malignant disease. The study protocol was reviewed and approved by the Ethics Committee of the Second Xiangya Hospital of Central South University. Prior to patient enrollment, we obtained written informed consent from either the patient or the patient’s authorized delegate (his/her next of kin or legal guardian) when the patient was comatose.

### Sample Collection

Plasma specimens were obtained from the patients with sepsis as soon as possible within 24 hours after their enrollment in the study. Peripheral blood was collected into EDTA tubes and centrifuged at 3000 g at 4°C for 10 min; then, the isolated plasma was frozen at -80°C within 1 h after collection.

### Data Collection

The demographic characteristics and clinical data, including age, gender, etiology of sepsis, infection sites, comorbidities at admission and final outcomes, were recorded for each subject. The vital signs, including temperature, blood pressure, heart rate, respiratory rate, and peripheral blood oxygen saturation, were recorded. The APACHE II score and the SOFA score were recorded as the lowest value obtained within the first 24 hours after enrollment. We also recorded the lengths of ICU stay and of hospital stay.

### Measurements

At the time of enrollment, coagulation system function; the serum lactate, bilirubin, PCT and creatinine levels; WBC count; and N% were routinely investigated. The CIRP plasma concentrations were measured in duplicate using a sandwich-based enzyme-linked immunosorbent assay (ELISA; CUSABIO, Wuhan, China), and the average concentrations of the duplicates were used for the analysis.

### Statistical Analysis

The results of the continuous variables are described as the means and standard deviations (SDs) or as the medians and inter quartile ranges (IQRs). Student’s t-test was used for the normally distributed data, and the Mann-Whitney U-test was used for the non-normally distributed data. Fisher’s exact test was used for the comparison of the categorical variables. Furthermore, we used Spearman rank correlation coefficients to assess the correlation of the CIRP level with the PCT level and with the WBC count. To evaluate the predictive value of the biomarkers for sepsis and the ability of the model to distinguish the survivor group from the nonsurvivor group, we generated receiver operating characteristic (ROC) curves and calculated the areas under the ROC curves (AUCs). A Cox proportional hazards regression model generated via forward stepwise selection procedures was used to identify the risk factors for hospital mortality. The variables with a *p*-value < 0.05 based on univariate analysis were entered into the multivariate model. All of the analyses were performed using IBM SPSS Statistics 21.0 for Microsoft Windows (IBM Corporation, Armonk, New York, United States). A *p*-value < 0.05 was considered to be statistically significant.

## Results

### Baseline Characteristics

Sixty-nine patients with sepsis were enrolled in this study. Their baseline demographic data are summarized in Table 1. According to the hospital mortality data, there were 38 survivors and 31 nonsurvivors. The differences in the age and sex distribution between the survivors and the nonsurvivors were not statistically significant (*p* = 0.659 and *p* = 0.552, respectively). The main sources of infection among the sepsis cases were pulmonary, abdominal, intracranial, skin and soft-tissue, urinary tract, and blood stream infections, in that order. In both groups, the primary comorbidities were diabetes, cardiovascular disease, hypertension, cerebrovascular disease, chronic pulmonary disease, post-operation, trauma, and others. There were no significant differences between the survivors and the nonsurvivors regarding these comorbidities (*p* = 0.484, *p* = 0.759, *p* = 0.969, *p* = 0.807, *p* = 0.653, *p* = 0.969, *p* = 0.228, and *p* = 0.726, respectively), whereas the APACHE II score was significantly higher in the nonsurvivors than in the survivors (median 27.0 versus 16.5; *p* = 0.000). Furthermore, in terms of organ function, the nonsurvivors had a higher SOFA score than the survivors (median 10 versus 6; *p* = 0.001). The length of ICU stay of the nonsurvivors was slightly longer than that of the survivors (mean 17.48 versus 10.71; *p* = 0.193), but there was no apparent difference in the length of hospital stay between these two groups (mean 24.39 versus 23.71; *p* = 0.907).

**Table 1:** Baseline demographics, clinical characteristics, and comorbidities of 69 patients with sepsis.

	Survivors (*n* = 38)	Nonsurvivors (*n* = 31)	*p-*value
**Age, years, mean (SD)**	58.3 (16.7)	60.1 (17.5)	0.659
**Male sex, *n* (%)**	27 (71.1)	24 (77.4)	0.552
**APACHE II score, median (IQR)**	16.5 (11.8–22.3)	27.0 (17.0–36.0)	0.000^a^
**Infection sources, *n* (%)**			
** Pulmonary**	23 (60.5)	24 (77.4)	0.195
** Abdominal**	11 (28.9)	3 (9.7)	0.071
** Intracranial**	1 (2.6)	3 (9.7)	0.319
** Skin and soft-tissue**	0 (0)	1 (3.2)	0.449
** Urinary tract**	1 (2.6)	0 (0)	1.000
** Bloodstream**	2 (5.3)	0 (0)	0.498
**Comorbidities, *n* (%)**			
** Diabetes**	5 (13.2)	6 (19.4)	0.525
** Cardiovascular disease**	4 (10.5)	4 (12.9)	1.000
** Hypertension**	6 (15.8)	5 (16.1)	1.000
** Cerebrovascular disease**	2 (5.3)	3 (9.7)	0.651
** Chronic pulmonary disease**	5 (13.2)	3 (9.7)	0.722
** Post-operation**	6 (15.8)	5 (16.1)	1.000
** Trauma**	6 (15.8)	2 (6.5)	0.281
** Others**	18 (47.4)	16 (51.6)	0.811
**Length of ICU stay, days, mean (SD)**	10.7 (8.3)	17.5 (27.4)	0.193
**Length of hospital stay, days, mean (SD)**	24.4 (14.8)	23.7 (32.0)	0.907
**SOFA score, median (IQR)**	6 (4–8)	10 (6–14)	0.001

APACHE II = Acute Physiology and Chronic Health Evaluation II, SOFA = Sepsis-related Organ Failure Assessment.

The *p*-values for age were calculated using the t-test, and those for the APACHE II scores and SOFA score were calculated using the Mann-Whitney *U* test. Fisher’s exact tests were applied for the categorical variables. A *p*-value < 0.05 was considered to be statistically significant.

IQR = inter-quartile range, SD = standard deviation.

^a^ *p* = 0.000433.

### Plasma Biomarker Levels

The plasma concentrations of CIRP and several other biomarkers are shown in Table 2. In our study, the median CIRP level in the nonsurvivors was significantly higher than that in the survivors (median (IQR) 4.99 (2.37–30.17) ng/mL versus 1.68 (1.41–13.90) ng/mL; *p* = 0.013). Additionally, the lactate and creatinine levels both showed significant differences between the nonsurvivor group and the survivor group (median (IQR) 1.90 (1.20–4.10) mmol/L versus 1.20 (0.70–1.73) mmol/L; *p* = 0.002; median (IQR) 124.40 (81.50–315.00) mmol/L versus 83.80 (55.90–123.05) mmol/L; *p* = 0.039). We were not able to distinguish the survivors from the nonsurvivors according to the WBC count, N%, or the PCT level (*p* = 0.708, *p* = 0.814, and *p* = 0.937, respectively).

**Table 2:** Comparison of the plasma biomarker levels between the survivors and nonsurvivors of sepsis.

	Survivors (*n* = 38)	Nonsurvivors (*n* = 31)	*p*-value
**CIRP (ng/mL), median (IQR)**	1.68 (1.41–13.90)	4.99 (2.37–30.17)	0.013
**WBC (×10** ^**9**^ **/L), median (IQR)**	11.91(8.88–16.96)	13.74(8.70–17.10)	0.708
**N%, median (IQR)**	89.28(86.71–92.66)	89.60(86.74–93.70)	0.814
**PCT (ng/mL), median (IQR)**	1.79(0.49–12.95)	2.38(0.56–9.70)	0.937
**Lactate (mmol/L), median (IQR)**	1.20(0.70–1.73)	1.90(1.20–4.10)	0.002
**Creatinine (mmol/L), median (IQR)**	83.80(55.90–123.05)	124.40(81.50–315.00)	0.039

CIRP = cold-inducible RNA-binding protein, WBC = white blood cell, N% = neutrophil ratio, PCT = procalcitonin.

The *p-*values for these biomarkers were obtained using the Mann-Whitney *U* test. A *p*-value < 0.05 was considered to be statistically significant.

IQR = inter-quartile range, SD = standard deviation.

### Correlation Analysis

**Fig 1:**
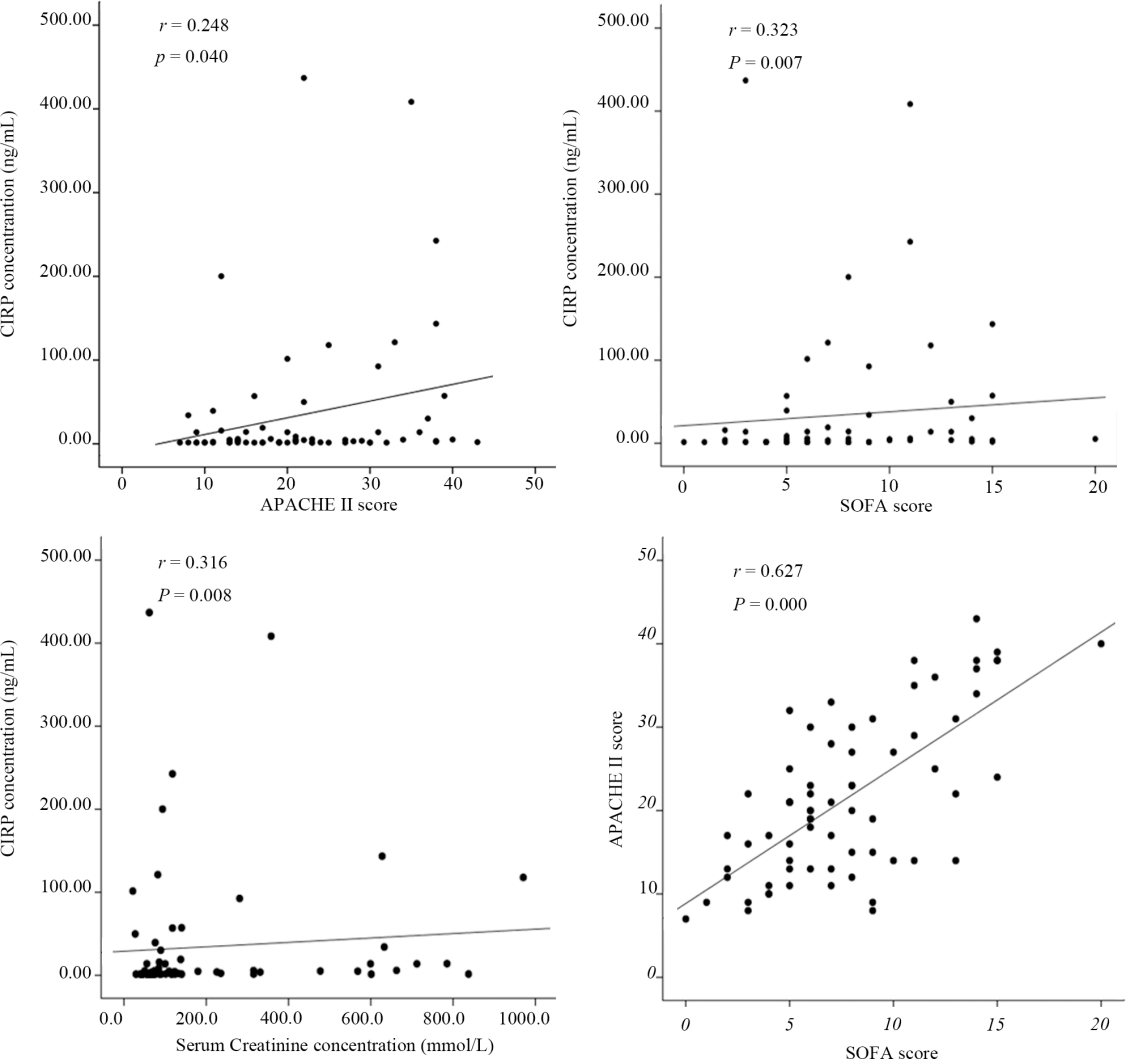
Correlations between the CIRP level and the levels of other biomarkers. The correlations of the plasma CIRP level with the APACHE II score; the SOFA score; the serum lactate, creatinine, and procalcitonin (PCT) levels; the white blood cell (WBC) count; and the neutrophil ratio (N%) were determined in the 69 patients with sepsis (Spearman rank analysis). *r* represents Spearman’s correlation coefficient, and a *p-*value < 0.05 was considered to be statistically significant.

The correlations between the CIRP level and the levels of other biomarkers of sepsis are shown in Fig 1. The correlations between the CIRP level and the APACHE II score (*r* = 0.248, *p* = 0.040, *n* = 69), the SOFA score (*r* = 0.323, *p* = 0.007, *n* = 69), the serum creatinine level (*r* = 0.316, *p* = 0.008, *n* = 69), and the PCT level (*r* = 0.282, *p* = 0.019, *n* = 69) were significant, whereas no significant correlations were found between the CIRP level and the lactate level (*r* = 0.230, *p* = 0.057, *n* = 69), the WBC count (*r* = -0.022, *p* = 0.857, *n* = 69), or the N% (*r* = -0.070, *p* = 0.566, *n* = 69). Interestingly, although there was no significant difference in the distribution of the PCT level in terms of outcomes, correlation analysis showed that the CIRP and PCT levels were significantly correlated (*r* = 0.282, *p* = 0.019, *n* = 69). Furthermore, there was a significant correlation between the SOFA score and the APACHE II score (*r* = 0.627, *p* = 0.000, *n* = 69). In addition, there were no significant correlations between the CIRP level and the length of ICU stay or the length of hospital stay.

### CIRP Level Predicts the Mortality of Patients with Sepsis

**Fig 2:**
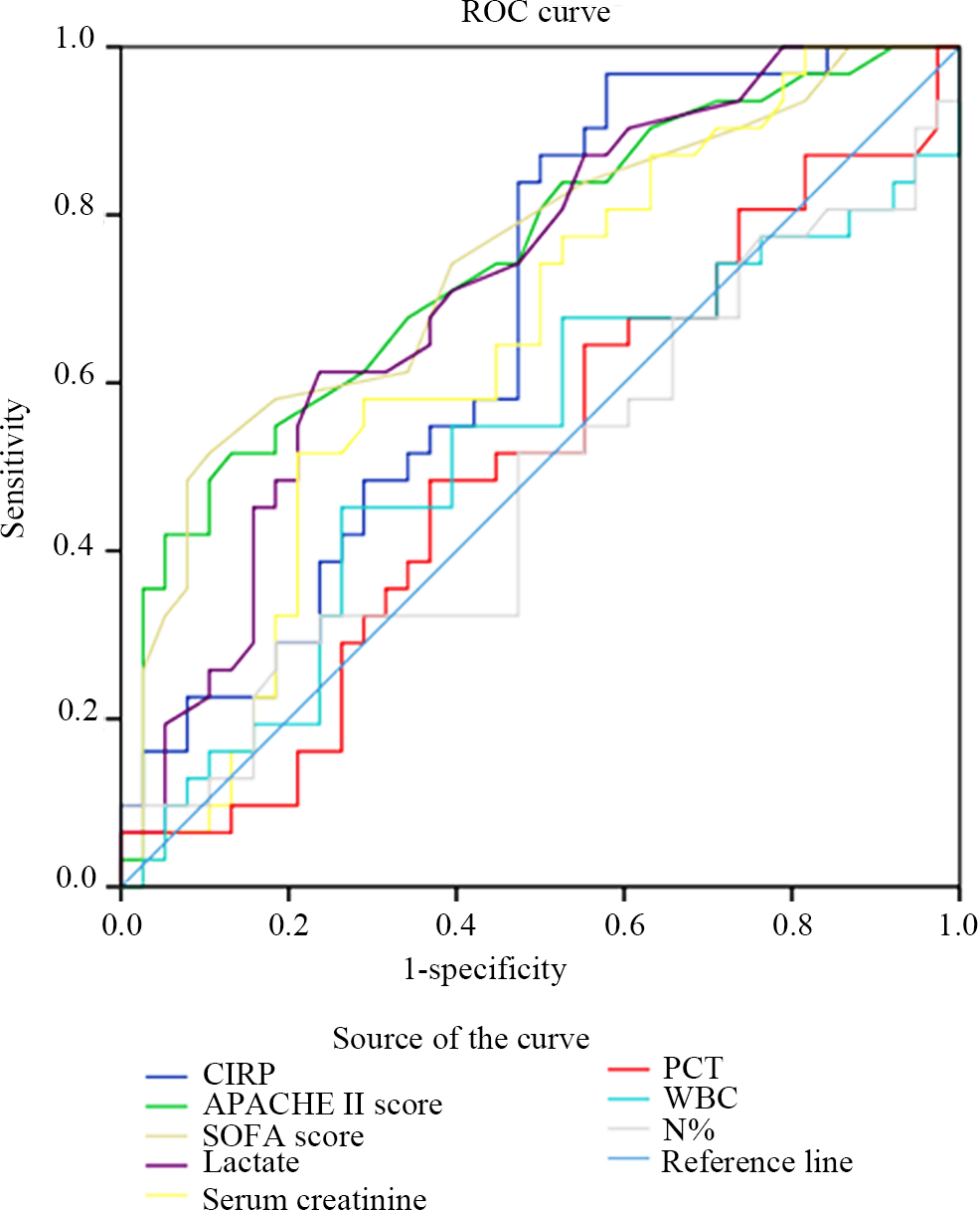
The ROC curves for the biomarkers and the severity scores. The areas under the ROC curve (AUCs) for the CIRP level, the APACHE II score, the SOFA score, the lactate level, the serum creatinine level, the PCT level, the WBC count, and the N% are shown.

First, we used ROC curve analysis to assess the predictive value of CIRP and the other biomarkers or parameters. As shown in Fig 2 and Table 3, the AUC for the CIRP level was 0.674 (95% CI, 0.547–0.801; *p* = 0.013); the AUCs of the APACHE II score, the SOFA score, the lactate level, and the serum creatinine level were 0.747 (95% CI, 0.631–0.864; *p* = 0.000), 0.743 (95% CI, 0.625–0.862; *p* = 0.001), 0.717 (95% CI, 0.597–0.837; *p* = 0.002), and 0.646 (95% CI, 0.515–0.776; *p* = 0.039), respectively. The optimal cutoff value of the CIRP level was 1.49 ng/mL, which displayed a sensitivity of 96.77% and a specificity of 42.11% for the prediction of mortality. Moreover, the optimal cutoff values of the other parameters were 26.00 for the APACHE II score, 9.50 for the SOFA score, 1.75 mmol/L for the lactate level, 124.1 mmol/L for the serum creatinine level, 3.25 ng/mL for the PCT level, 15.83×10^9^/L for the WBC count, and 93.34% for the N%. Table 3 shows the statistical data for these parameters based on their respective thresholds.

**Table 3:** Mortality prediction based on the plasma levels of the biomarkers and on the severity scores according to ROC curve analysis.

Prediction for mortality	CIRP level	APACHE II score	SOFA score	Lactate level	Serum creatinine	PCT level	WBC count	N%
**Optimal cutoff-value**	1.49 ng/mL	26.00	9.50	1.75 mmol/L	124.10 mmol/L	3.25 ng/mL	15.83×10^9^/L	93.34%
**Sensitivity, (%) (95%CI)**	96.77 (83.30–99.92)	51.61 (33.06–69.85)	51.61 (33.06–69.85)	61.29 (42.19–78.15)	51.61 (33.06–69.85)	48.39 (30.15–66.94)	45.16 (27.32–63.97)	29.03 (14.22–48.04)
**Specificity, (%) (95%CI)**	42.11 (26.31–59.18)	86.84 (71.91–95.59)	89.47 (75.20–97.06)	76.32 (59.76–88.56)	78.95 (62.68–90.45)	63.16 (45.99–78.19)	73.68 (56.90–86.60)	81.58 (65.67–92.26)
**Positive likelihood ratio**	1.67	3.92	4.90	2.59	2.45	1.31	1.72	1.58
**Negative likelihood ratio**	0.08	0.56	0.54	0.51	0.61	0.82	0.74	0.87
**Positive predictive value (%)**	57.69	76.19	80.00	67.86	66.67	51.72	58.33	56.25
**Negative predictive value (%)**	94.12	68.75	69.39	70.73	66.67	60.00	62.22	58.49
**AUC**	0.674	0.747	0.743	0.717	0.646	0.506	0.526	0.483
***p*- value**	0.013	0.000^b^	0.001	0.002	0.039	0.938	0.708	0.814

The optimal cutoff values for each plasma biomarker level and the severity scores are presented. A *p*-value < 0.05 was considered to be statistically significant.

CIRP = cold-inducible RNA-binding protein, APACHE II = Acute Physiology and Chronic Health Evaluation II, SOFA = Sequential Organ Failure Assessment score, PCT = procalcitonin, WBC = white blood cell, N% = neutrophil ratio.

ROC = receiver operating characteristic, AUC = area under the ROC curve, CI = confidence interval.

^b^ *p* = 0.000437.

A multivariate Cox regression was used for univariate analysis and hazard ratio calculation. The results are presented in Table 4. Because the distribution of the CIRP concentrations was broad, data transformation was performed. CIRP0.1 was used to represent the value of the CIRP concentration divided by 10. The results showed that CIRP0.1 and the lactate level were independently associated with mortality according to the examined parameters. The hazard ratio was 1.05 (95% CI, 1.01–1.09) for CIRP0.1 and 1.26 (95% CI, 1.10–1.45) for the lactate level.

**Table 4:** Cox proportional hazards models for mortality prediction according to the biomarker levels and the severity scores.

Variable	Univariate Cox model		Multivariable Cox model	
	HR(95%CI)	*p*-value	HR(95%CI)	*p*-value
**CIRP0.1**	1.05(1.01–1.08)	0.009	1.05(1.01–1.09)	0.012
**APACHE II score**	1.05(1.01–1.09)	0.007	1.05(0.99–1.11)	0.112
**SOFA score**	1.10(1.01–1.21)	0.028	0.98(0.84–1.13)	0.742
**Lactate**	1.18(1.05–1.34)	0.008	1.26(1.10–1.45)	0.001
**Serum creatinine**	1.00(1.00–1.00)	0.940	1.00(1.00–1.00)^c^	0.820
**PCT**	1.00(0.99–1.02)	0.426	1.00(0.98–1.02)	0.821
**WBC**	1.01(0.96–1.07)	0.594	1.024(0.98–1.08)	0.339
**N%**	0.97(0.93–1.00)	0.061	0.96(0.92–1.00)	0.037

CIRP = cold-inducible RNA-binding protein, APACHE II = Acute Physiology and Chronic Health Evaluation II, SOFA = Sepsis-related Organ Failure Assessment, PCT = procalcitonin, WBC = white blood cell, N% = neutrophil ratio.

CIRP0.1 = CIRP level/10.

HR = hazard ratio.

A *p*-value < 0.05 was considered to be statistically significant.

^c^ = 0.998–1.002

### CIRP Level Predicts Renal Injury in Patients with Sepsis

We compared the biomarker levels and the severity scores using AUCs to predict the severity of sepsis and multiple-organ dysfunction (Table 5). In patients with sepsis, the APACHE II score, the SOFA score, the lactate level, the serum creatinine concentration, and the PCT level can partially predict the severity of sepsis or organ dysfunction. The CIRP level reflected the degree of renal injury (AUC = 0.653) but did not predict the severity of sepsis or damage to other organs.

**Table 5:** Areas under the receiver operating characteristic curves for certain biomarker levels and the severity scores in the prediction of sepsis severity and organ failure in patients with sepsis.

	CIRP level	APACHE II score	SOFA score	Lactate level	Serum creatinine level	PCT level	WBC count	****N%****
**Septic shock**	0.479	0.704*	0.705*	0.632	0.490	0.608	0.447	0.489
**Severe sepsis**	0.600	0.608	0.722**	0.971**	0.710**	0.755**	0.544	0.526
**Coagulopathy**	0.523	0.715*	0.833**	0.613	0.874**	0.746**	0.513	0.428
**Hyperbilirubinemia**	0.665	0.452	0.617	0.358	0.358	0.615	0.392	0.437
**Creatinine elevation**	0.653*	0.641	0.787**	0.643	0.947**	0.810**	0.544	0.400
**Thrombocytopenia**	0.583	0.598	0.767**	0.686*	0.658*	0.711**	0.437	0.490

*p-value ≦ 0.05

**p-value ≦ 0.01

## Discussion

The baseline demographics indicate that the survivor and the nonsurvivor groups were highly comparable. The differences in the sources of infection and in the distribution of the comorbidities between the two groups were not significant. The question remains regarding what types of patients with sepsis are most likely to die. In the field of critical care medicine, there are two classic scoring systems: the APACHE II score, which is used to evaluate disease severity, and the SOFA score, which is used to assess organ dysfunction [25, 26]. In this observational cohort study, these two scores were significantly higher in the nonsurvivors than in the survivors. However, ROC curve analysis revealed that the sensitivities of these two scoring systems were only approximately 50% in predicting mortality among the patients with sepsis. Therefore, neither of these scoring systems is suitable for mortality risk screening. For the same reason, the lactate, serum creatinine, and PCT levels; the WBC count; and the N% are also unsuitable as screening indicators. Interestingly, no differences in the lengths of ICU stay or of hospital stay were observed between these two groups. These results may be related to several non-medical factors. First, in our hospital, some of the patients had to remain in the ICU because there was no common ward available, even if their condition improved. Second, some of the patients who could have been discharged were unwilling to leave a tertiary care hospital because of imperfections in the local referral system.

A complex, biologically mediated network is the basis for the clinical manifestations of sepsis [27, 28]. CIRP is a stress response protein, and previous reports in the literature have revealed that its presence is related to the inflammation caused by the stress of sepsis [23]. Macrophages play important roles in immunomodulation and immune defense [29, 30]. CIRP in the circulatory system originates from macrophages under hypoxic stress, and recombinant CIRP can induce macrophages to release the proteins tumor necrosis factor-α (TNF-α) and high mobility group box 1 (HMGB1), which are proinflammatory factors that exacerbate the damage to organ function [23]. Hypoxic stress can induce the expression, translocation and release of CIRP, and extracellular CIRP has been confirmed to intensify inflammation and cause organ damage [12, 20, 22, 23].

An optimal biomarker may serve one or more of the following overlapping functions: screening, diagnosis, risk stratification, monitoring, surrogate assessment and point-of-care [31]. This study found that the plasma level of CIRP was not only significantly different between the nonsurvivor group and the survivor group, although the CIRP level displayed high sensitivity for the prediction of sepsis mortality. CIRP levels greater than 1.49 ng/mL displayed very high sensitivity (96.15%), a very low negative likelihood ratio (0.09) and a very high negative predictive value (94.2%); the AUC for the CIRP level was 0.674 for sepsis mortality (*p* = 0.013). These results suggest that CIRP can be used as a screening tool for the early prediction of the mortality risk of sepsis. An elevated plasma concentration of CIRP was associated with increased mortality. Multivariate Cox regression analysis showed that when the plasma CIRP level increased by 10 ng/mL, the mortality risk increased by 1.05-fold. CIRP is an independent risk factor for mortality in sepsis (*p* = 0.012), second only to lactate (*p* = 0.001), and more significantly than the APACHE II and SOFA scores (*p* = 0.112 and *p* = 0.742, respectively). The CIRP and lactate levels reflect the degree of inflammation and tissue perfusion, respectively, both of which are key determinants of prognosis.

Compared with other classic indicators, the CIRP level reflected the degree of renal injury but did not predict the severity of sepsis or damage to other organs. Because of this disappointing result, we believe that studies using larger sample sizes are needed to confirm these results and to perform further analysis. Further clarification of the role of CIRP in the pathogenesis of sepsis will provide a theoretical basis for these findings.

## Limitations

There were many limitations to this study. First, we used the peripheral blood concentration of CIRP as a surrogate for the total CIRP concentration in the body, without considering that the CIRP concentration may be different in specific organs and tissues. Although there have been no human studies related to this issue, several animal studies have shown that a variety of tissues and organs exhibit CIRP expression [32, 33]. Second, only one test was performed within 24 hours after enrollment, without determining whether the CIRP concentration changes over time as a patient’s condition fluctuates. Many sepsis patients experience deterioration in their condition after 24 hours or longer, depending on when they were hospitalized. Third, we did not include non-septic patients in this study; thus, we were unable to determine whether the observed elevated plasma concentrations of CIRP were specific to sepsis. Fourth, some of the enrolled patients may have been misdiagnosed with sepsis and may have ultimately developed etiologies of other diseases. Fifth, this was a single-center study, and our sample size was not large. The findings of this study must be confirmed by further multi-center, large-sample clinical studies in the future.

## Conclusions

In patients with sepsis, elevated plasma concentrations of CIRP are significantly associated with poor prognosis. Risk stratification, which is performed to implicate distinct prognoses, can help physicians to identify the patients who may exhibit poor outcomes, as such patients may warrant particular attention. CIRP is a promising biomarker for predicting the prognosis of sepsis. According to the most recent research, targeting CIRP can protect the liver from ischemia-reperfusion injury [20]. As CIRP has been demonstrated to act as a potent inflammatory mediator in sepsis, we hypothesize that blocking CIRP protects against inflammatory injury and improves patient outcomes [26]. This will be the direction of our future research.

## Supporting Information

S1 File Relevant data underlying the findings described in manuscript. (XLSX)

## References

[1] Dellinger RP, Levy MM, Rhodes A, Annane D, Gerlach H, Opal SM, et al Surviving sepsis campaign: international guidelines for management of severe sepsis and septic shock: 2012. Critical care medicine. 2013;41(2):580–637. 10.1097/CCM.0b013e31827e83af . 23353941

[2] Caironi P, Tognoni G, Masson S, Fumagalli R, Pesenti A, Romero M, et al Albumin replacement in patients with severe sepsis or septic shock. The New England journal of medicine. 2014;370(15):1412–21. 10.1056/NEJMoa1305727 . 24635772

[3] Asfar P, Meziani F, Hamel JF, Grelon F, Megarbane B, Anguel N, et al High versus low blood-pressure target in patients with septic shock. The New England journal of medicine. 2014;370(17):1583–93. 10.1056/NEJMoa1312173 . 24635770

[4] Holst LB, Haase N, Wetterslev J, Wernerman J, Guttormsen AB, Karlsson S, et al Lower versus higher hemoglobin threshold for transfusion in septic shock. The New England journal of medicine. 2014;371(15):1381–91. 10.1056/NEJMoa1406617 . 25270275

[5] Pro CI, Yealy DM, Kellum JA, Huang DT, Barnato AE, Weissfeld LA, et al A randomized trial of protocol-based care for early septic shock. The New England journal of medicine. 2014;370(18):1683–93. 10.1056/NEJMoa1401602 24635773 PMC4101700

[6] Investigators A, Group ACT, Peake SL, Delaney A, Bailey M, Bellomo R, et al Goal-directed resuscitation for patients with early septic shock. The New England journal of medicine. 2014;371(16):1496–506. 10.1056/NEJMoa1404380 . 25272316

[7] Barrett ML, Smith MW, Elixhauser A, Honigman LS, Pines JM. Utilization of Intensive Care Services, 2011: Statistical Brief #185. Healthcare Cost and Utilization Project (HCUP) Statistical Briefs. Rockville (MD)2014.

[8] Jiang L, Feng B, Gao D, Zhang Y. Plasma concentrations of copeptin, C-reactive protein and procalcitonin are positively correlated with APACHE II scores in patients with sepsis. The Journal of international medical research. 2015;43(2):188–95. 10.1177/0300060514561136 . 25691533

[9] Kim H, Kim Y, Lee HK, Kim KH, Yeo CD. Comparison of the delta neutrophil index with procalcitonin and C-reactive protein in sepsis. Clinical laboratory. 2014;60(12):2015–21. . 25651736 10.7754/clin.lab.2014.140528

[10] Umberger R, Thompson CL, Cashion AK, Kuhl D, Wan J, Yates CR, et al Exaggerated plasma interleukin 6, interleukin 10, and subsequent development of health care-associated infections in patients with sepsis. Dimensions of critical care nursing: DCCN. 2015;34(2):100–11. 10.1097/DCC.0000000000000098 . 25650495

[11] Nishiyama H, Itoh K, Kaneko Y, Kishishita M, Yoshida O, Fujita J. A glycine-rich RNA-binding protein mediating cold-inducible suppression of mammalian cell growth. The Journal of cell biology. 1997;137(4):899–908. 9151692 10.1083/jcb.137.4.899 PMC2139845

[12] Sakurai T, Kashida H, Watanabe T, Hagiwara S, Mizushima T, Iijima H, et al Stress response protein cirp links inflammation and tumorigenesis in colitis-associated cancer. Cancer research. 2014;74(21):6119–28. 10.1158/0008-5472.CAN-14-0471 . 25187386

[13] Artero-Castro A, Callejas FB, Castellvi J, Kondoh H, Carnero A, Fernandez-Marcos PJ, et al Cold-inducible RNA-binding protein bypasses replicative senescence in primary cells through extracellular signal-regulated kinase 1 and 2 activation. Molecular and cellular biology. 2009;29(7):1855–68. 10.1128/MCB.01386-08 19158277 PMC2655622

[14] Sakurai T, Kudo M, Watanabe T, Itoh K, Higashitsuji H, Arizumi T, et al Hypothermia protects against fulminant hepatitis in mice by reducing reactive oxygen species production. Digestive diseases. 2013;31(5–6):440–6. 10.1159/000355242 . 24281018

[15] Tong G, Endersfelder S, Rosenthal LM, Wollersheim S, Sauer IM, Buhrer C, et al Effects of moderate and deep hypothermia on RNA-binding proteins RBM3 and CIRP expressions in murine hippocampal brain slices. Brain research. 2013;1504:74–84. 10.1016/j.brainres.2013.01.041 . 23415676

[16] Brochu C, Cabrita MA, Melanson BD, Hamill JD, Lau R, Pratt MA, et al NF-kappaB-dependent role for cold-inducible RNA binding protein in regulating interleukin 1beta. PloS one. 2013;8(2):e57426 10.1371/journal.pone.0057426 23437386 PMC3578848

[17] Liu Y, Hu W, Murakawa Y, Yin J, Wang G, Landthaler M, et al Cold-induced RNA-binding proteins regulate circadian gene expression by controlling alternative polyadenylation. Scientific reports. 2013;3:2054 10.1038/srep02054 23792593 PMC3690385

[18] Jiang D, Zhu XL, Zhao JF, Zhou YK, Zhong C, Zhang J, et al Subtractive screen of potential limb regeneration related genes from Pachytriton brevipes. Molecular biology reports. 2014;41(2):1015–26. 10.1007/s11033-013-2946-z . 24390235

[19] Xia ZP, Zheng XM, Zheng H, Liu XJ, Liu GY, Wang XH. Downregulation of cold-inducible RNA-binding protein activates mitogen-activated protein kinases and impairs spermatogenic function in mouse testes. Asian journal of andrology. 2012;14(6):884–9. 10.1038/aja.2012.71 23001445 PMC3720101

[20] Godwin A, Yang WL, Sharma A, Khader A, Wang Z, Zhang F, et al Blocking cold-inducible RNA-binding protein protects liver from ischemia-reperfusion injury. Shock. 2015;43(1):24–30. 10.1097/SHK.0000000000000251 25186836 PMC4270919

[21] Das U. HLA-DR expression, cytokines and bioactive lipids in sepsis. Archives of medical science: AMS. 2014;10(2):325–35. 10.5114/aoms.2014.42586 24904669 PMC4042054

[22] Zhou M, Yang WL, Ji Y, Qiang X, Wang P. Cold-inducible RNA-binding protein mediates neuroinflammation in cerebral ischemia. Biochimica et biophysica acta. 2014;1840(7):2253–61. 10.1016/j.bbagen.2014.02.027 24613680 PMC4061249

[23] Qiang X, Yang WL, Wu R, Zhou M, Jacob A, Dong W, et al Cold-inducible RNA-binding protein (CIRP) triggers inflammatory responses in hemorrhagic shock and sepsis. Nature medicine. 2013;19(11):1489–95. 10.1038/nm.3368 24097189 PMC3826915

[24] Levy MM, Fink MP, Marshall JC, Abraham E, Angus D, Cook D, et al 2001 SCCM/ESICM/ACCP/ATS/SIS International Sepsis Definitions Conference. Critical care medicine. 2003;31(4):1250–6. 10.1097/01.CCM.0000050454.01978.3B . 12682500

[25] Knaus WA, Draper EA, Wagner DP, Zimmerman JE. APACHE II: a severity of disease classification system. Critical care medicine. 1985;13(10):818–29. . 3928249

[26] Vincent JL, Moreno R, Takala J, Willatts S, De Mendonca A, Bruining H, et al The SOFA (Sepsis-related Organ Failure Assessment) score to describe organ dysfunction/failure. On behalf of the Working Group on Sepsis-Related Problems of the European Society of Intensive Care Medicine. Intensive care medicine. 1996;22(7):707–10. . 8844239 10.1007/BF01709751

[27] Cheng B, Hoeft AH, Book M, Shu Q, Pastores SM. Sepsis: pathogenesis, biomarkers, and treatment. BioMed research international. 2015;2015:846935 10.1155/2015/846935 25834828 PMC4365376

[28] Pop-Began V, Paunescu V, Grigorean V, Pop-Began D, Popescu C. Molecular mechanisms in the pathogenesis of sepsis. Journal of medicine and life. 2014;7 Spec No. 2:38–41. 25870671 PMC4391358

[29] Na YR, Yoon YN, Son D, Jung D, Gu GJ, Seok SH. Consistent inhibition of cyclooxygenase drives macrophages towards the inflammatory phenotype. PloS one. 2015;10(2):e0118203 10.1371/journal.pone.0118203 25680189 PMC4334507

[30] Liu YC, Zou XB, Chai YF, Yao YM. Macrophage polarization in inflammatory diseases. International journal of biological sciences. 2014;10(5):520–9. 10.7150/ijbs.8879 24910531 PMC4046879

[31] Marshall JC, Reinhart K, International Sepsis F. Biomarkers of sepsis. Critical care medicine. 2009;37(7):2290–8. 10.1097/CCM.0b013e3181a02afc . 19487943

[32] Sakurai T, Yada N, Watanabe T, Arizumi T, Hagiwara S, Ueshima K, et al Cold-inducible RNA-binding protein promotes the development of liver cancer. Cancer science. 2015;106(4):352–8. 10.1111/cas.12611 . 25611373 PMC4409877

[33] Tang C, Wang Y, Lan D, Feng X, Zhu X, Nie P, et al Analysis of gene expression profiles reveals the regulatory network of cold-inducible RNA-binding protein mediating the growth of BHK-21 cells. Cell biology international. 2015 10.1002/cbin.10438 . 25597958

